# Effects of applying platelet-rich plasma during arthroscopic rotator cuff repair: a systematic review and meta-analysis of randomised controlled trials

**DOI:** 10.1038/s41598-020-74341-0

**Published:** 2020-10-14

**Authors:** Fu-An Yang, Chun-De Liao, Chin-Wen Wu, Ya-Chu Shih, Lien-Chen Wu, Hung-Chou Chen

**Affiliations:** 1 School of Medicine, College of Medicine, Taipei Medical University, Taipei, Taiwan; 2 Department of Physical Medicine and Rehabilitation, Shuang Ho Hospital, Taipei Medical University, No. 291 Jhongjheng Road, Jhonghe District, New Taipei City, 235 Taiwan; 3 School and Graduate Institute of Physical Therapy, College of Medicine, National Taiwan University, Taipei, Taiwan; 4 Department of Physical Medicine and Rehabilitation, School of Medicine, College of Medicine, Taipei Medical University, Taipei, Taiwan; 5 Department of Orthopedics, School of Medicine, College of Medicine, Taipei Medical University, Taipei, Taiwan; 6 Department of Orthopedics, Shuang Ho Hospital, Taipei Medical University, New Taipei City, Taiwan; 7 Center for Evidence-Based Health Care, Shuang Ho Hospital, Taipei Medical University, New Taipei City, Taiwan

**Keywords:** Musculoskeletal abnormalities, Tendons, Orthopaedics

## Abstract

Because of its healing properties, platelet-rich plasma (PRP) has been applied to the bone–tendon interface during arthroscopic rotator cuff repair to improve surgical outcomes. However, its effects remain ambiguous. Therefore, we conducted this systematic review and meta-analysis to assess the effects of PRP on retear rate and functional outcomes. Randomised control trials were identified and extracted. Data collection was completed on 15 February 2020. The results are expressed as the risk ratio (RR) for the categorical variables and weighted mean difference for the continuous variables, with 95% confidence intervals (CIs). Analyses were performed using RevMan 5.3 software. Seven randomised controlled trials published from 2013 to 2018, with 541 patients in total, were included. The results revealed a significant decrease in retear rate [RR 0.38, 95% CI (0.22, 0.68), *P* = 0.0009). Furthermore, a significant improvement was observed regarding short-term Constant score [mean difference = 3.28, 95% CI (1.46, 5.11), *P* = 0.0004), short-term University of California at Los Angeles activity score [mean difference = 1.60, 95% CI (0.79, 2.42), *P* = 0.0001], and short-term visual analogue scale score [mean difference =  − 0.14, 95% CI (− 0.23, − 0.05), *P* = 0.002]. This systematic review indicates the efficacy of PRP when applied to the bone–tendon interface during arthroscopic rotator cuff repair.

## Introduction

Rotator cuff tear causes pain and limited motor function of shoulder^1,2^. The gold standard for treatment is arthroscopic repair when conventional treatment fails^3,4^. Even though the surgical technique has been continually improved, the retear rate remains high at 34%–94%, as revealed by several studies^5–8^. Notably, poor tendon-bone healing is considered a cause of retear^9,10^. The original fibrocartilage tissue is replaced by fibrovascular scar tissue after surgery, which has lower mechanical flexibility than does the original tissue^11–14^. Besides, pain and weakness are also an issue of rotator cuff tear which may have a great influence on the patients’ daily activity. It is worth mentioning that several studies have demonstrated the benefits of platelet-rich plasma (PRP) therapy as an adjuvant to rotator cuff repair to improve surgical outcomes^15–20^.

PRP is a concentration of platelets prepared through centrifugation of autologous whole blood^21^. It contains an abundance of growth factors, such as platelet-derived growth factor (PDGF), transforming growth factor (TGF)-beta, fibroblast growth factor (FGF), vascular endothelial growth factor (VEGF), and epidermal growth factor (EGF)^22,23^. Recent studies have reported that PRP can potentially aid wound repair^10,11,22^. Nevertheless, the effects of PRP on rotator cuff repair remain controversial^15–17^. Delve into the previous studies^15–17^, some of these^15,16^ stated that PRP application is effective in reducing retear rate and improving clinical outcome while the other^17^ showed no improvement in both. Furthermore, some studies included different type of rotator cuff tear, some included PRP application both intra-operative and post-operative, and some included patients with rotator cuff tear that were not diagnosed by MRI or sonography pre-operative. Hence, we conducted this systematic review and meta-analysis of randomised controlled trials (RCTs) to evaluate the outcome of PRP application on the bone–tendon interface during arthroscopic rotator cuff repair.

## Methods

### Inclusion and exclusion criteria

The inclusion criteria of this study were as follows: (1) full-thickness rotator cuff tear; (2) diagnosis based on MRI or sonography; (3) application of arthroscopic rotator cuff repair; (4) application of PRP on the bone–tendon interface during arthroscopic repair in the intervention group; and (5) reported outcomes, including retear rate (defined following Sugaya et al.^24^ as type IV or V for MRI findings and following Barth et al.^25^ as grade IV or V for sonography findings), Constant score, the University of California at Los Angeles (UCLA) activity score, Disabilities of the Arm, Shoulder, and Hand (DASH) score, or visual analogue scale (VAS) score.

The exclusion criteria of the study were as follows: (1) application of a plasma-rich fibrin or matrix; (2) diagnosis not based on MRI or sonography findings; (3) partial-thickness tear of the rotator cuff; and (4) sonography-guided injection of PRP postoperatively.

### Search strategy

The authors independently screened the literature, extracted data, and performed crosschecks in accordance with the Preferred Reporting Items for Systematic Reviews and Meta-Analyses guidelines^26^. We searched electronic databases, such as PubMed, EMBASE, Cochrane, and Google Scholar. Medical subject heading (MeSH) terms were used for searching these electronic databases, and ‘Platelet-Rich Plasma’ (MeSH) AND ‘Rotator Cuff’ (MeSH) were used as the keywords. RCTs were identified using the refined search function in the databases, if available. In addition, articles were identified through a manual search of the reference lists of the relevant articles. The literature search spanned the date of database inception to 15 February 2020. Two reviewers independently reviewed the full texts of all potentially relevant articles to identify articles meeting the eligibility criteria. The individually recorded decisions of the two reviewers were then compared, and dissimilarities in the decisions were resolved by a third reviewer.

### Data items

The following information was obtained from each RCT identified: type of rotator cuff tear, image used for diagnosis, surgical procedure, number and mean age of the participants of PRP and control groups, follow-up duration, follow-up images, and outcome measurements.

### Risk-of-bias assessment

Risk-of-bias assessment was performed using the RoB 2 tool, a revised Cochrane risk-of-bias tool for randomised trials, which is a widely used quality assessment tool for evaluating RCTs^27^. The following domains were assessed: (1) randomisation process, (2) deviations from intended interventions, (3) missing outcome data, (4) outcome measurement, (5) selection of the reported result, and (6) overall bias. Risk-of-bias assessment was conducted by two independent reviewers according to Cochrane Handbook for Systematic Reviews of Interventions^28^. Differences of opinion between reviewers were resolved by discussion and consultation with a third author.

### Statistical analysis

Statistical analysis was performed using RevMan 5.3 software, which was provided by the Cochrane Collaboration (https://training.cochrane.org/online-learning/core-software-cochrane-reviews/revman/revman-5-download). A *P* value of < 0.05 was considered statistically significant. We used the *I*^*2*^ test to provide an objective measurement of statistical heterogeneity. According to the Cochrane Handbook for Systematic Reviews of Interventions^28^, heterogeneity was quantified using the *I*^*2*^ statistic with a rough guide for interpretation as follows: 0–40%—might not be important, 30–60%—may represent moderate heterogeneity, 50–90%—may represent substantial heterogeneity, and 75–100%—considerable heterogeneity. A random-effects model was used in this meta-analysis. As for the result with *I*^*2*^ > 50% even though a random-effects model was used, we will remove the study that result in the heterogeneity. The results were expressed as the risk ratio (RR) for the categorical variables and as the weighted mean difference for the continuous variables, with 95% confidence intervals (CIs). Because of differences in surgical patterns, a subgroup analysis was performed on the basis of surgical pattern (single-row and double-row repair). Furthermore, we used a cut-off point of 12 months for determining whether differences existed between short-term (≤ 12 months) and long-term (> 12 months) follow-ups.

Notably, a funnel plot was not used to test publication bias because of the limited number (< 10) of studies included in each analysis.

## Results

### Search results

**Figure 1:**
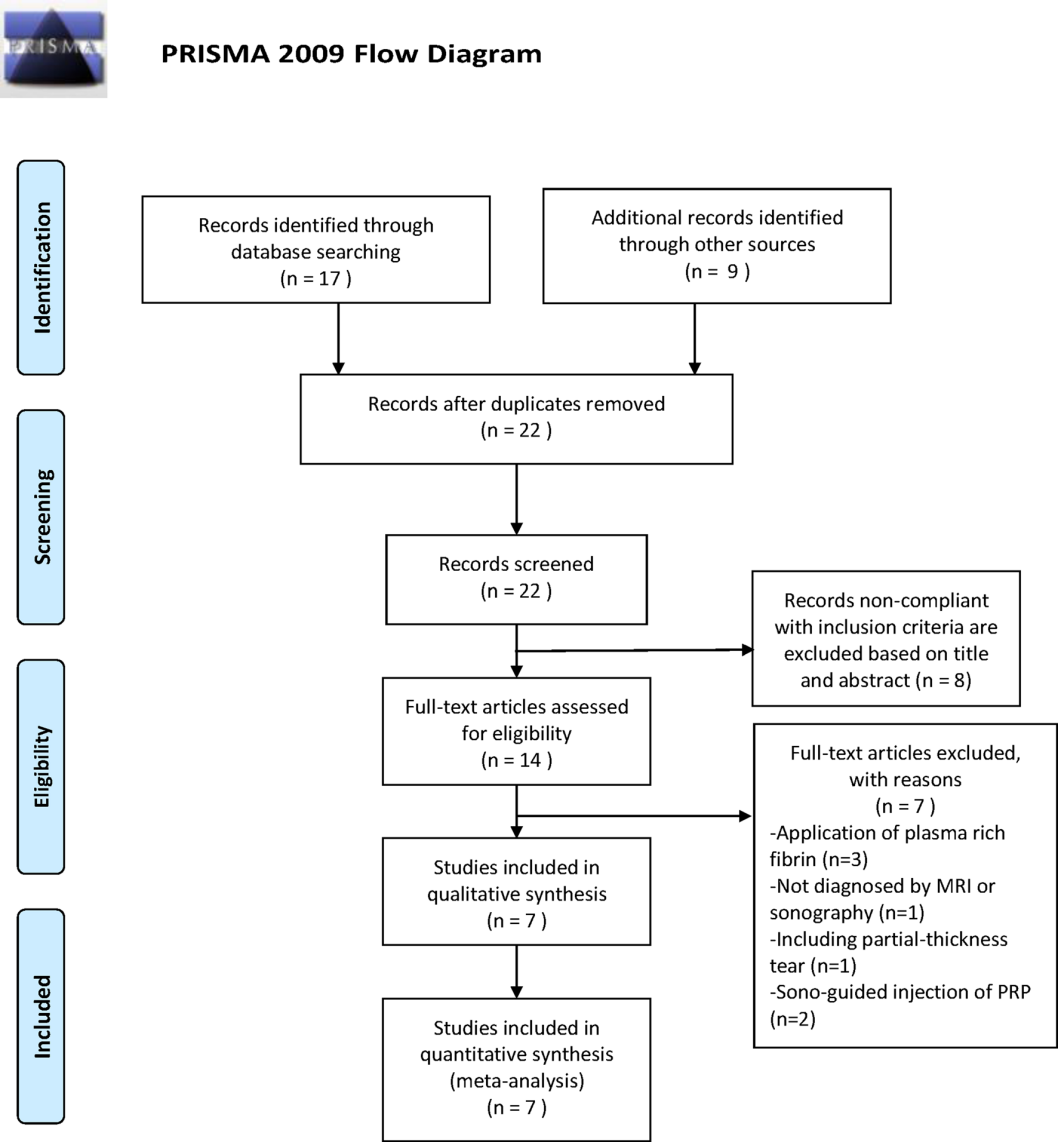
Flow chart showed detail information for article inclusion and exclusion (From Moher D, Liberati A, Tetzlaff J, Altman DG, The PRISMA Group (2009). Preferred reporting items for systematic reviews and meta-analyses: the PRISMA statement. PLoS Med 6(7): e1000097. 10.1371/journal.pmed1000097).

When we used the previously stated search terms, 26 RCTs were initially retrieved. Four duplicates were excluded using EndNote X9^29^. Eight citations that were noncompliant with the inclusion criteria were excluded after their title and abstract were screened. The full text of the remaining 14 citations was screened, which revealed three articles that dealt with the use of plasma-rich fibrin, one that involved diagnosis not based on MRI or sonography findings, one that dealt with partial-thickness tear, and two that involved PRP application through sonography-guided injection postoperatively. Finally, seven articles were selected for this systematic review and meta-analysis^30–36^ (Fig. 1).

### Study characteristics

All studies included were published between 2013 and 2018 and included 541 patients (273 patients in the PRP group and 268 in the control group). Only one study^31^ involved diagnosis based on MRI or sonography findings, whereas all others used MRI for diagnosis^30,32–36^. Three studies^30–32^ involved single-row repair, and four^33–36^ involved double-row repair. The main characteristics of the seven RCTs included are summarised in Table 1.

**Table 1:** Characteristics of the included randomised control trials (RCTs).

Author, year	Rotator cuff tear	Diagnosed	Procedure	PRP group		Control group		Follow-up (months)	Follow-up image	Outcome
				n	mean age (SD)	n	mean age (SD)			
Eduardo Angeli Malavolta, 2014^30^	Complete supraspinatus tear (< 30 mm)	MRI	Single-row repair	27	55.3 (8.3)	27	54.07 (6.59)	24	MRI	(1), (2), (3), (5)
Vivek Pandey, 2016^31^	Full-thickness medium to large rotator cuff tear	MRI or US	Single-row repair	56	54.8 (8.4)	54	54.1 (8.3)	24	US	(1), (2), (3), (5)
Eduardo Angeli Malavolta, 2018^32^	Complete supraspinatus tear (> 30 mm)	MRI	Single-row repair	39	54 (6.5)	36	55.4 (8.4)	60	MRI	(1), (2), (3), (5)
Chris Hyunchul Jo, 2013^33^	Large to massive rotator cuff tear (> 30 mm)	MRI	Double-row repair	24	64.21 (6.09)	24	61.92 (8.36)	12	MRI and CTA	(1), (2), (3), (4), (5)
Chris Hyunchul Jo, 2015^34^	Median to large rotator cuff tear (> 10 mm, < 50 mm)	MRI	Double-row repair	37	60.08 (4.88)	37	60.92 (7.34)	12	MRI	(1), (2), (3), (5)
Zhenxiang ZHANG, 2016^35^	Full-thickness rotator cuff tear (> 10 mm)	MRI	Double-row repair	30	56.9 (6)	30	57.2 (7.4)	12	MRI	(1), (2), (4), (5)
Matthias Flury, 2016^36^	Complete rotator cuff tear of the supraspinatus tendon	MRI	Double-row repair	60	57.8 (8)	60	58.9 (8.2)	24	MRI or US	(1)
n total				273		268				

(1) Retear rate, 7 RCTs; (2) Constant score, 6 RCTs; (3) UCLA score, 5 RCTs; (4) DASH score, 2 RCTs; (5) VAS score, 6 RCTs.

*PRP* platelet-rich plasma, *MRI* magnetic resonance imaging, *US* ultrasonography, *UCLA score* University of California at Los Angeles activity score, *DASH score* disabilities of the arm, shoulder, and hand score, *VAS* visual analogue scale score.

### Risk-of-bias assessment

**Figure 2:**
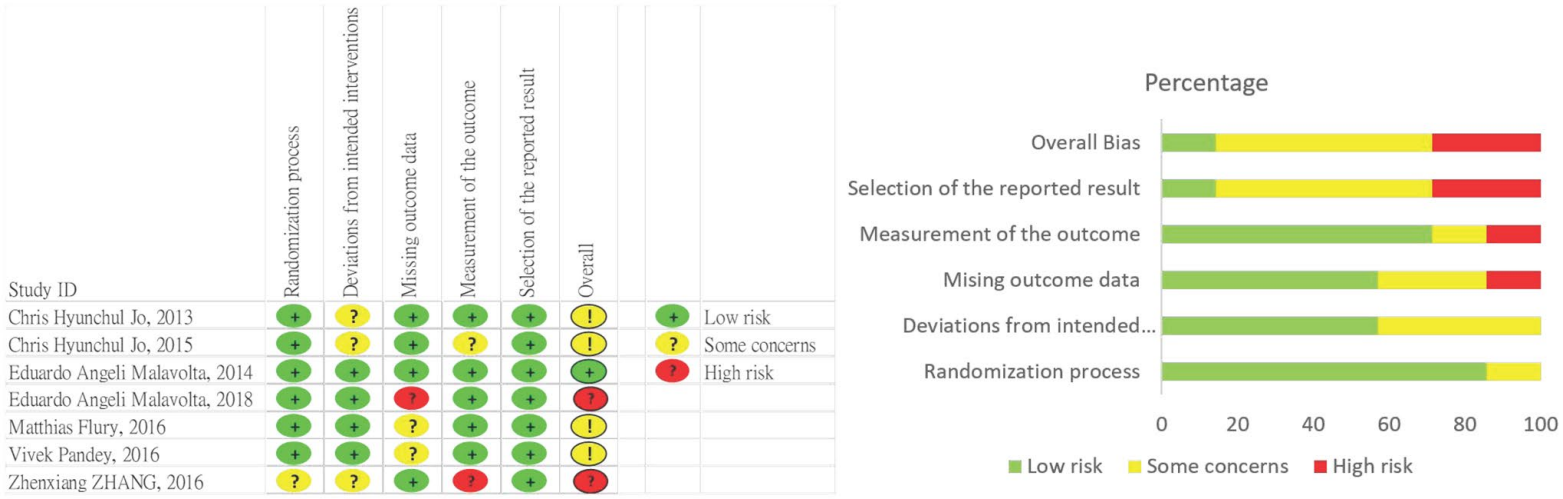
Quality assessment.

The quality of the RCTs included was assessed by two reviewers independently by using the RoB 2 tool, a revised Cochrane RoB tool for randomised trials^27^. The risk of bias in each study is illustrated in Fig. 2.

Six studies^30–34,36^ were identified as having low risk in the randomisation process, and one was identified as having uncertain risk^35^. The risk of deviations from intended interventions was low in four studies^30–32,36^ and uncertain in three studies^33–35^. Two studies were identified as having uncertain risk^31,36^ and was identified as having high risk^32^ related to missing outcome data. Furthermore, one study was high risk in terms of outcome measurement^35^. All studies had a low risk for selection of the reported result^30–36^. Finally, the risk of overall bias was noted as low in one study^30^, uncertain in four studies^31,33,34,36^, and high in two studies^32,35^.

### Retear rate

**Figure 3:**
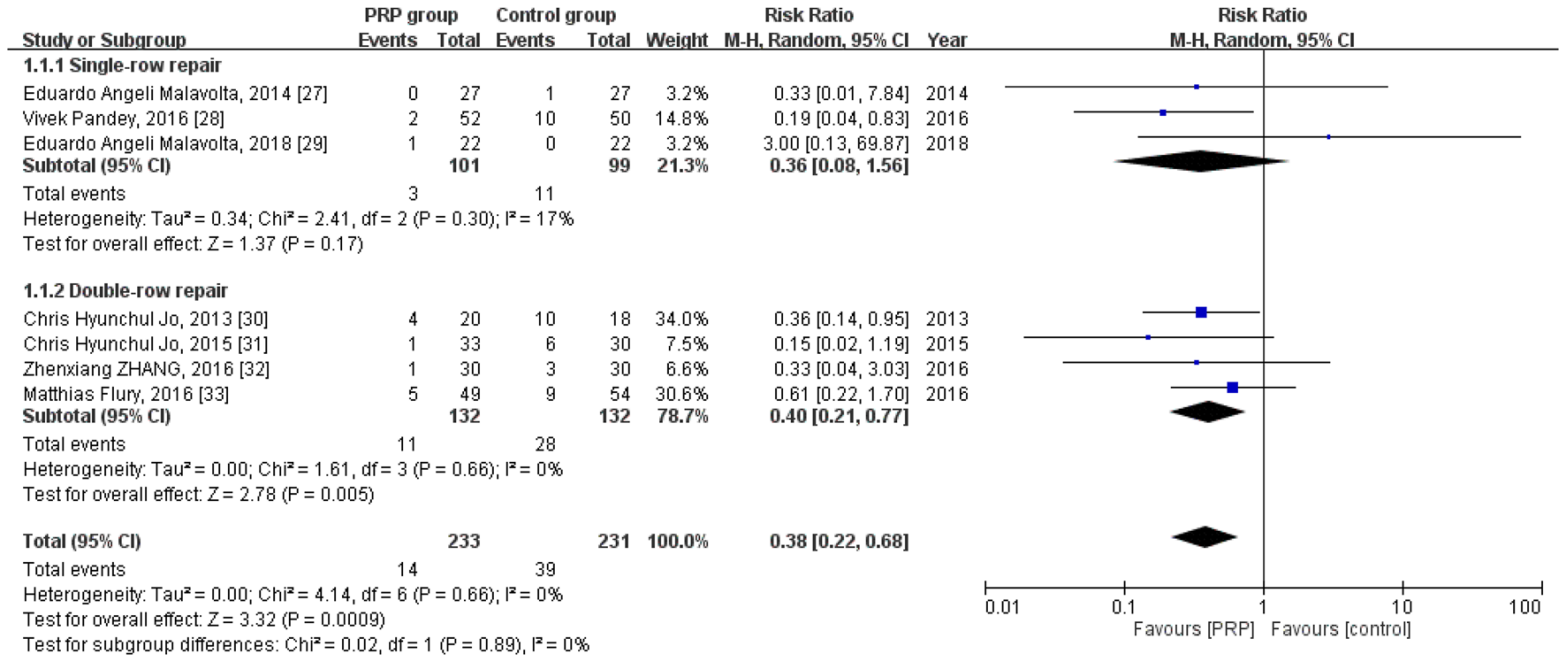
Forest plot for the re-tear rate.

Retear rate was reported by all seven studies^30–36^, which included 233 patients in the PRP group and 231 in the control group. The homogeneity across the studies was good (*I*^*2*^ = 0%, *P* = 0.66). The retear rate was significantly lower in the PRP group than in the control group [RR = 0.38, 95% CI (0.22, 0.68), *P* = 0.0009). Subgroup analysis revealed a statistically significant intergroup difference related to the retear rate for double-row repair [RR = 0.40, 95% CI (0.21, 0.77), *P* = 0.005] but not for single-row repair [RR = 0.36, 95% CI (0.08, 1.56), *P* = 0.17] (Fig. 3).

### Short-term constant score

**Figure 4:**
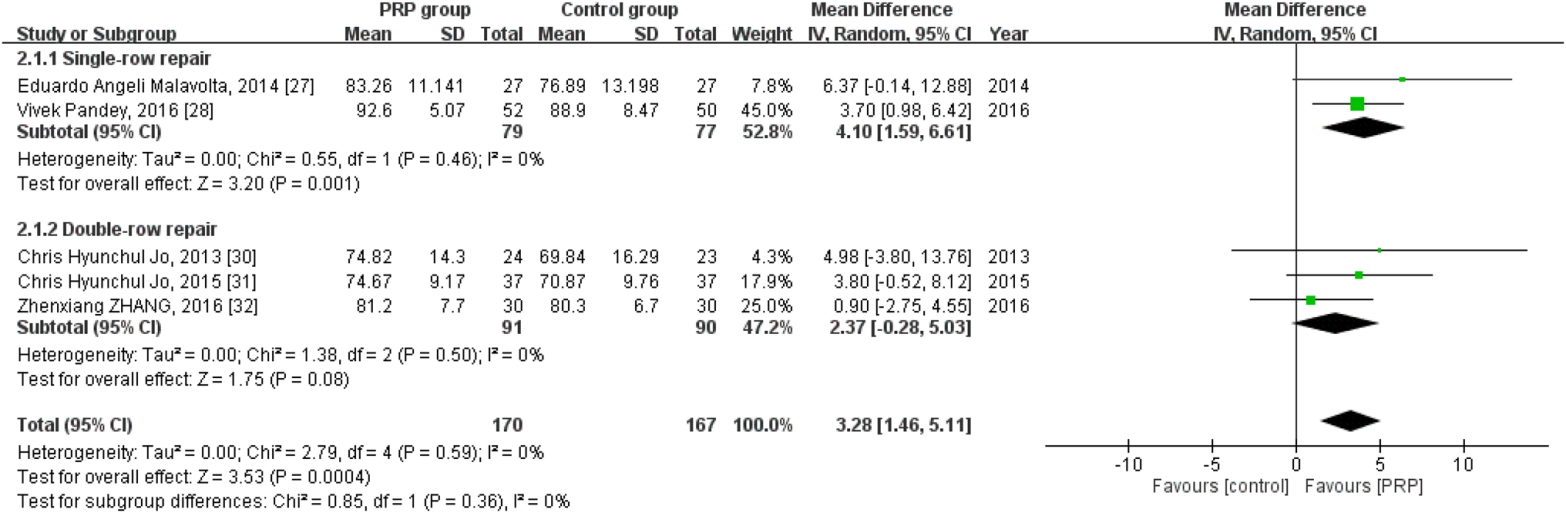
Forest plot for the short-term constant score.

Five studies reported the short-term Constant score^30,31,33–35^, with 170 patients in the PRP group and 167 in the control group. The homogeneity across the studies was good (*I*^*2*^ = 0%, *P* = 0.59). The short-term Constant score was significantly higher in the PRP group than in the control group [mean difference = 3.28, 95% CI (1.46, 5.11), *P* = 0.0004]. Moreover, subgroup analysis revealed a statistically significant intergroup difference related to the short-term Constant score for single-row repair [mean difference = 4.10, 95% CI (1.59, 6.61), *P* = 0.001] but not for double-row repair [mean difference = 2.37, 95% CI (− 0.28, 5.03), *P* = 0.08] (Fig. 4).

### Long-term constant score

**Figure 5:**
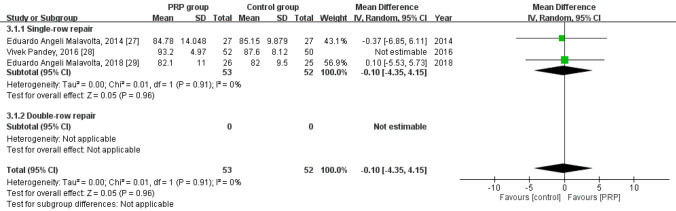
Forest plot for the long-term constant score.

Three studies reported the long-term Constant score^30–32^, with 105 patients in the PRP group and 102 in the control group. The heterogeneity across the studies was high (*I*^*2*^ = 60%, *P* = 0.08) and thus the study by Pandey et al.^31^ was removed, which reduced the value of *I*^*2*^ to 0%, indicating good homogeneity (*P* = 0.91). No statistically significant intergroup differences were observed with respect to the long-term Constant score [mean difference = -0.10, 95% CI (− 4.35, 4.15), *P* = 0.96] (Fig. 5).

### Short-term UCLA score

**Figure 6:**
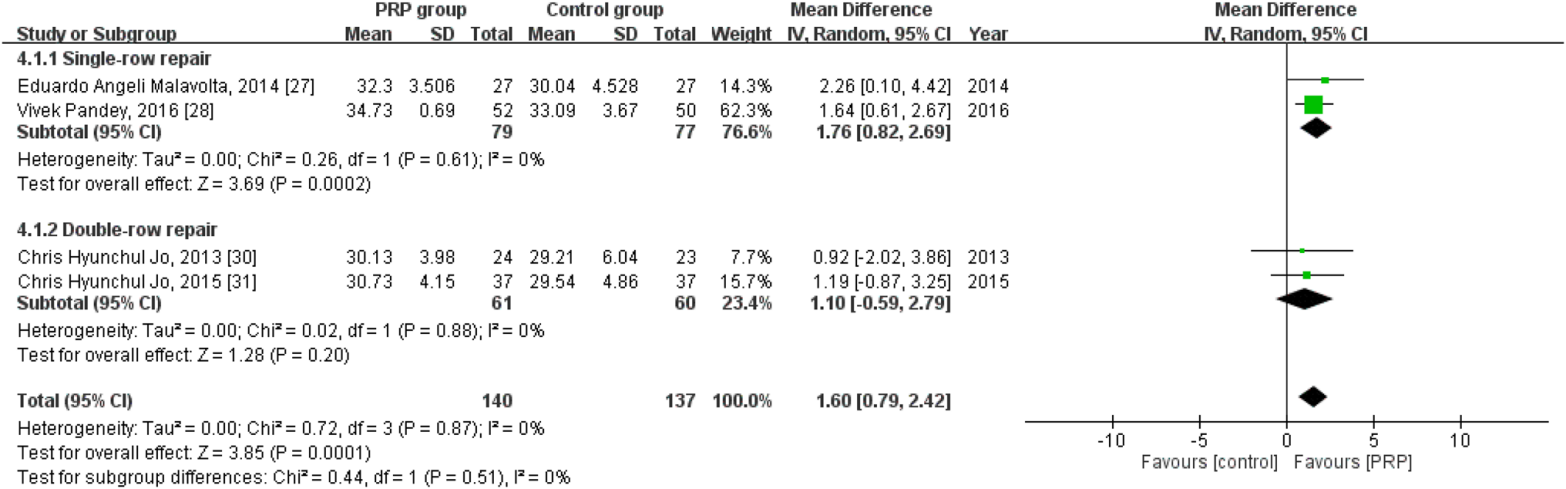
Forest plot for the short-term UCLA score.

Four studies reported the short-term UCLA score^30,31,33,34^, with 140 patients in the PRP group and 137 in the control group. The homogeneity across the studies was good (*I*^*2*^ = 0%, *P* = 0.87). The short-term UCLA score was significantly higher in the PRP group than in the control group [mean difference = 1.60, 95% CI (0.79, 2.42), *P* = 0.0001]. Subgroup analysis revealed a statistically significant intergroup difference in terms of short-term UCLA score for single-row repair [mean difference = 1.76, 95% CI (0.82, 2.69), *P* = 0.0002] but not for double-row repair [mean difference = 1.10, 95% CI (− 0.59, 2.79), *P* = 0.20] (Fig. 6).

### Long-term UCLA score

**Figure 7:**
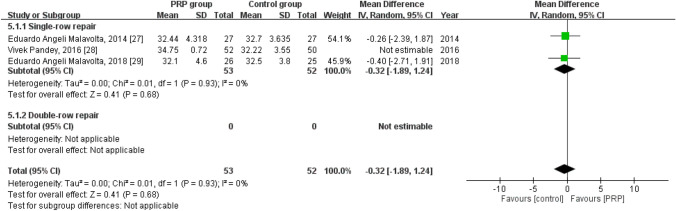
Forest plot for the long-term UCLA score.

Three studies reported the long-term UCLA score^30–32^, with 105 patients in the PRP group and 102 in the control group. The heterogeneity across the studies was high (*I*^*2*^ = 78%, *P* = 0.01) and thus the study by Pandey et al.^31^ was removed, which reduced the value of *I*^*2*^ to 0%, indicating good homogeneity (*P* = 0.93). No statistically significant intergroup differences were noted regarding the long-term UCLA score [mean difference = -0.32, 95% CI (− 1.89, 1.24), *P* = 0.68] (Fig. 7).

### Short-term DASH score

**Figure 8:**
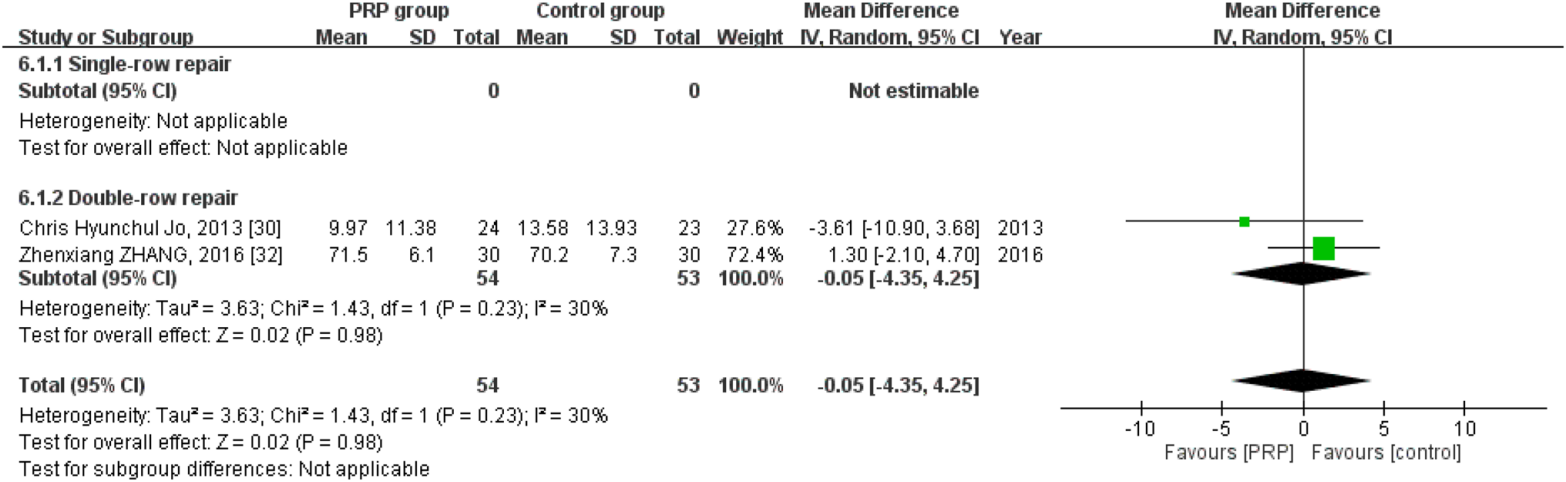
Forest plot for the short-term DASH score.

Two studies reported the short-term DASH score^33,34^, with 54 patients in the PRP group and 53 in the control group. The homogeneity across the studies was good (*I*^*2*^ = 30%, *P* = 0.23). No statistically significant intergroup differences were noted regarding the short-term DASH score [mean difference =  − 0.05, 95% CI (− 4.35, 4.25), *P* = 0.98] (Fig. 8).

**Figure 9:**
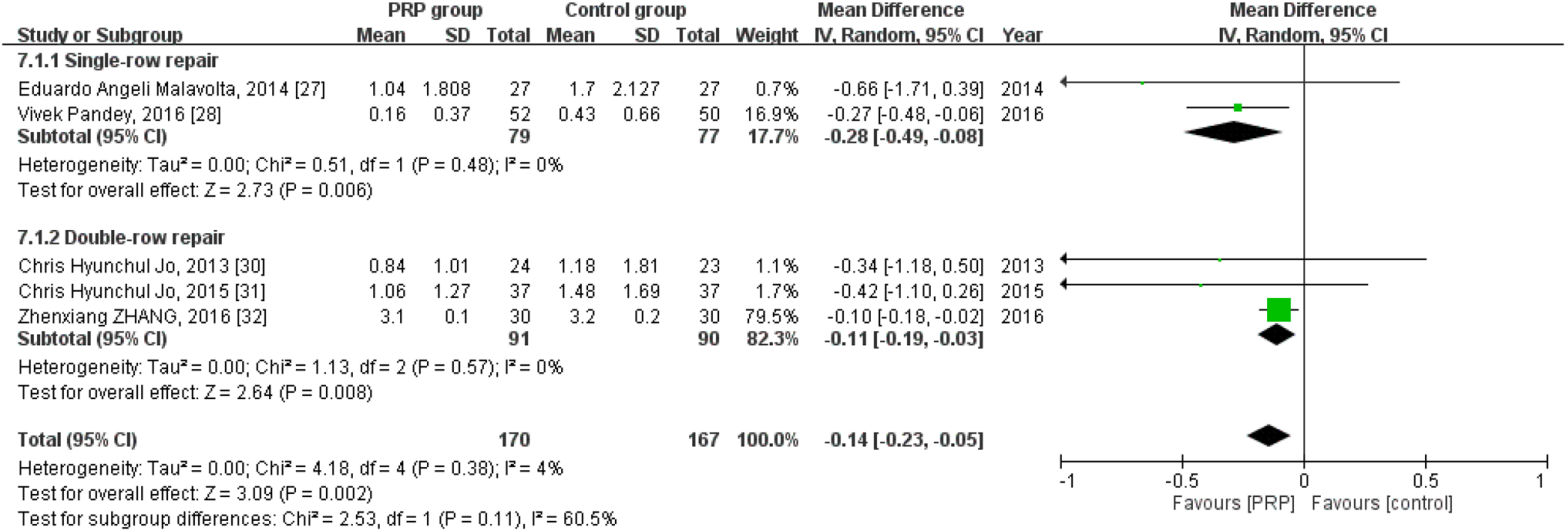
Forest plot for the short-term VAS score.

Short-term VAS score:

Five studies reported the short-term VAS score^30,31,33–35^, with 170 patients in the PRP group and 167 in the control group. The homogeneity across the studies was good (*I*^*2*^ = 4%, *P* = 0.38). The short-term VAS score was significantly lower in the PRP group than in the control group [mean difference =  − 0.14, 95% CI (− 0.23, − 0.05), *P* = 0.002]. Subgroup analysis revealed statistically significant intergroup difference with respect to the short-term VAS score for both the single-row [mean difference =  − 0.28, 95% CI (− 0.49, − 0.08)], *P* = 0.006] and double-row [mean difference =  − 0.11, 95% CI (− 0.19, − 0.03), *P* = 0.008] repair patterns (Fig. 9).

### Long-term VAS score

**Figure 10:**
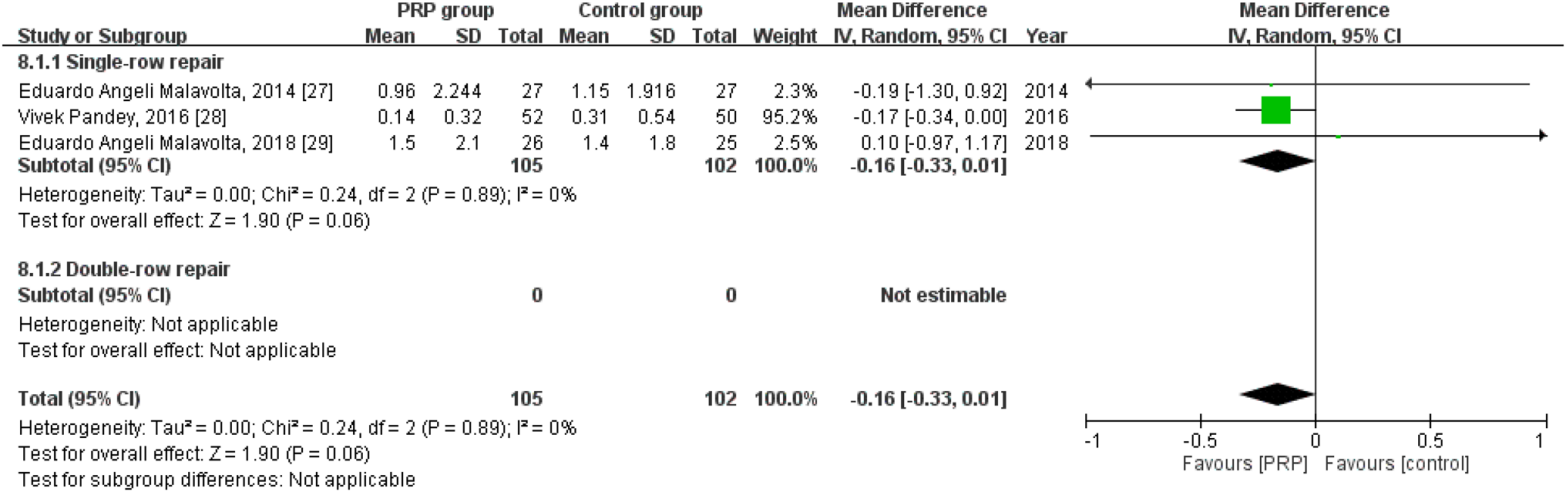
Forest plot for the long-term VAS score.

Three studies reported the long-term VAS score^30–32^, with 105 patients in the PRP group and 102 in the control group. The homogeneity across the studies was good (*I*^*2*^ = 0%,* P* = 0.89). No statistically significant intergroup differences were noted with respect to the long-term VAS score [mean difference =  − 0.16, 95% CI (− 0.33, 0.01), *P* = 0.06] (Fig. 10).

Table 2 is a summarization of the subgroup analysis.

**Table 2:** Summary of subgroup analysis.

Outcome	Single-row repair	Double-row repair	Overall
Retear rate (RR, 95% CI)	0.36 [0.08, 1.56]	0.40 [0.21, 0.77]*	0.38 [0.22, 0.68]*
Short-term Constant score (MD, 95% CI)	4.10 [1.59, 6.61]*	2.37 [− 0.28, 5.03]	3.28 [1.46, 5.11]*
Long-term Constant score (MD, 95% CI)	− 0.10 [− 4.35, 4.15]	–	− 0.10 [− 4.35, 4.15]
Short-term UCLA score (MD, 95% CI)	1.76 [0.82, 2.69]*	1.10 [− 0.59, 2.79]	1.60 [0.79, 2.42]*
Long-term UCLA score (MD, 95% CI)	− 0.32 [− 1.89, 1.24]	–	− 0.32 [− 1.89, 1.24]
Short-term DASH score (MD, 95% CI)	− 0.05 [− 4.35, 4.25]	–	− 0.05 [− 4.35, 4.25]
Short-term VAS score (MD, 95% CI)	− 0.28 [− 0.49, − 0.08]*	− 0.11 [− 0.19, − 0.03]*	− 0.14 [− 0.23, − 0.05]*
Long-term VAS score (MD, 95% CI)	− 0.16 [− 0.33, 0.01]	–	− 0.16 [− 0.33, 0.01]

‘*’ Shows statistical differences; ‘–’ means not assessable.

*UCLA score* University of California at Los Angeles activity score, *DASH score* disabilities of the arm, shoulder, and hand score, *VAS* visual analogue scale score, *RR* risk ratio, *MD* mean difference, *CI* confidence interval.

## Discussion

We conducted this systematic review and meta-analysis to determine the effects of applying PRP to the bone–tendon interface during arthroscopic rotator cuff repair. The results of our analysis revealed statistically significant differences in the following aspects:

1. Retear rate, for overall and double-row repair in subgroup analysis.
2. Short-term Constant score, for overall and single-row repair in subgroup analysis.
3. Short-term UCLA score, for overall and single-row repair in subgroup analysis.
4. Short-term VAS score, for overall and both single-row and double-row repair in subgroup analysis.

The primary goal of PRP use is to reduce the retear rate. The tendon healing process can be divided into three stages, namely inflammation, proliferation, and remodeling^37^. Notably, different growth factors are required to achieve this goal. PRP has been widely used to improve the healing of bones, cartilage, and tendons^21,38–41^. PRP contains an abundance of growth factors, such as PDGF, TGF-beta, FGF, VEGF, and EGF^22,23^. These growth factors can trigger tissue regeneration^42–44^ and improve the vascularity of the repaired tendon^45–48^, thereby reducing the retear rate. Our analysis revealed a statistically significant decrease in the retear rate of the PRP group, namely in the double-row repair subgroup.

According to our finding, there are significant differences in terms of short-term outcomes but not in that of long-term ones. Zumstein et al.^49^ stated that the single application of PRP is effective in increasing the local level of growth factors for up to 28 days, and that could trigger healing process of the repaired area. The injured tendon is stated to recover to its maximum strength in 12 months^50^. As a result, when added on PRP application, it shows significant better outcomes in short-term follow up, while the effect of PRP application is not obvious in long-term follow up.

Previous studies had demonstrated that double-row repair has better clinical outcomes when compared with single-row repair due to it provides a better biomechanical property ^51,52^, and thus, provides a better environment for tendon healing. As a result, the effect of PRP administration is not obvious. In contrast, single-row repair provides a lower biomechanical strength, and thus the effect of PRP administration is significant. It corresponds to our result which demonstrates better clinical outcomes in single-row repair group in the subgroup analysis for a short-term follow up. However, there is no adequate data to analysis the difference in each group of a long-term follow up. Thus, further studies comparing single-row and double-row repair for a long-term follow up are warranted.

Nevertheless, our review had several differences compared with previous systematic reviews and meta-analyses^15–17^. First, the previous reviews included studies that involved intraoperative administration and sonography-guided postoperative administration of PRP. Furthermore, each study has different times of administration sonography-guided postoperatively. We thought that it will result in different outcome when compared with intraoperative administration. As a result, our review included studies that dealt with PRP application on the bone–tendon interface during arthroscopic repair since it is stated by Zumstein et al.^49^ that the single application of PRP is effective in increasing the local level of growth factors for up to 28 days. Second, our review included studies that administrated only PRP and not any other platelet-rich matrix in order to lower the bias caused by different material applied. Third, all the RCTs included in this review were conducted on patients with full-thickness rotator cuff tear who received diagnoses based on preoperative MRI or sonography findings since other studies included participants of partial tear and those diagnosed intraoperatively.

Nonetheless, our review has some limitations. First, the absence of a standard preparation of PRP and the use of varying concentrations and amounts of PRP in the studies may have caused different outcomes in individual studies. Second, the tear size differed among studies. Third, the sample size of the included studies was relatively small and the data is not adequate for further analysis for long-term follow up in different surgical type. Thus, further reviews involving high-quality, large-scale RCTs are needed to overcome the limitations of this review.

## Conclusion

This systematic review and meta-analysis revealed that application of PRP to the bone–tendon interface during arthroscopic rotator cuff repair is beneficial. PRP was observed to reduce the retear rate and improve functional outcomes, namely during the short-term follow-up of single-row repair. In other words, we recommend the application of PRP on bone-tendon interface during arthroscopic rotator cuff repair for the improvement of patients’ early functional outcomes, especially in single-row repair. Last but not the least, further high-quality and large-scale RCTs are needed to provide more information of the benefit of PRP.
